# A longitudinal investigation into cognition and disease progression in spinocerebellar ataxia types 1, 2, 3, 6, and 7

**DOI:** 10.1186/s13023-016-0447-6

**Published:** 2016-06-22

**Authors:** Amy Moriarty, Arron Cook, Helen Hunt, Matthew E. Adams, Lisa Cipolotti, Paola Giunti

**Affiliations:** Department of Neuropsychology, National Hospital for Neurology and Neurosurgery, Queen Square, London, WC1N 3BG UK; Department of Molecular Neuroscience, Ataxia Centre, UCL Institute of Neurology, Queen Square, London, WC1N 3BG UK; Lysholm Department of Neuroradiology, National Hospital for Neurology and Neurosurgery, Queen Square, London, WC1N 3BG UK; Dipartimento Di Psicologia, Universita Degli Studi Di Palermo, Palermo, Italy

**Keywords:** Ataxia, Spinocerebellar ataxia, Cognition

## Abstract

**Background:**

The natural history of clinical symptoms in the spinocerebellar ataxias (SCA)s has been well characterised. However there is little longitudinal data comparing cognitive changes in the most common SCA subtypes over time. The present study provides a preliminary longitudinal characterisation of the clinical and cognitive profiles in patients with SCA1, SCA2, SCA3, SCA6 and SCA7, with the aim of elucidating the role of the cerebellum in cognition.

**Methods:**

13 patients with different SCAs all caused by CAG repeat expansion (SCA1, n = 2; SCA2, n = 2; SCA3, n = 2; SCA6, n = 4; and SCA7, n = 3) completed a comprehensive battery of cognitive and mood assessments at two time points, a mean of 7.35 years apart. All patients were evaluated clinically using the Scale for the Rating and Assessment of Ataxia (SARA) and the Inventory of Non-Ataxia Signs (INAS). Patients underwent structural MRI imaging at follow-up.

**Results:**

Clinical scale scores increased in all patients over time, most prominently in the SCA1 (SARA) and SCA3 (INAS) groups. New impairments on neuropsychological tests were most commonly observed with executive functions, speed, attention, visual memory and Theory of Mind. Results suggest possible differences in cognitive decline in SCA subtypes, with the most rapid cognitive decline observed in the SCA1 patients, and the least in the SCA6 patients, congruent with observed patterns of motor deterioration. Minimal changes in mood were observed, and MRI measures of atrophy did not correlate with cognitive decline.

**Conclusion:**

As well as increasing physical impairment, cognitive decline over time appears to be a distinct aspect of the SCA phenotype, in keeping with the cerebellar cognitive-affective syndrome. Our data suggest a trend of cognitive decline that is different for each SCA subtype, and for the majority is related to the severity of cerebellar motor impairment.

**Electronic supplementary material:**

The online version of this article (doi:10.1186/s13023-016-0447-6) contains supplementary material, which is available to authorized users.

## Background

The spinocerebellar ataxias (SCAs) are a clinically and genetically heterogeneous group of dominantly inherited neurodegenerative disorders, with a prevalence range of 0.9-3 in 100,000 [1]. Over thirty genetic subtypes have been described; the most common of these are SCA1, SCA2, SCA3, SCA6 and SCA7 [2]. The SCAs are associated with a polyglutamine repeat expansion, the length of which is inversely correlated to the age of onset of symptoms [1, 3]. In SCA1, SCA2, SCA3 and SCA7, symptoms typically develop in the fourth decade, whereas in SCA6 onset is approximately 20 years later [4]. Whilst the cardinal clinical features of the SCAs involve gait and truncal ataxia, dysarthria and limb ataxia, phenotypic differences include diplopia, progressive visual loss, spasticity, dysphagia, parkinsonism, dystonia, peripheral neuropathy and urge incontinence [5]. The progression of cerebellar symptoms in the SCAs has been well characterised. A large two-year follow-up study involving over 400 patients reported the greatest annual increase in ataxia severity amongst SCA1 patients, followed by SCA3 and SCA2 patients [6]. In contrast, SCA6 patients exhibited a slower, non-linear pattern of progression. The findings for non-ataxia signs were similar; the fastest rate of decline was described for SCA1 while SCA2 and SCA3 were slower but similar. Some non-ataxia signs were present in SCA6 but no progression was observed over the two-year period. Polyglutamine repeat length and age of onset were associated with the more rapid clinical progression in SCA1 and SCA2 patients. In SCA3, clinical progression was affected by disease duration. In a second study involving a sub-group of the same sample, clinical progression was assessed in the context of MRI-based volumetry [7]. This study demonstrated a correlation between ataxia symptoms and brainstem atrophy in SCA1 and SCA3. In addition, cerebellar atrophy was also associated with symptoms in SCA3. A comparison of clinical progression in SCA7 patients amongst other SCA groups has yet to be completed, but this phenotype is known to involve retinal degeneration and progressive visual loss [1, 8].

Cognitive impairments have been described in many SCA subtypes and patterns of impairment vary considerably [9–11]. Deficits in attention, executive functions, visuoconstructive skills, visual and verbal memory and processing speed have all been reported. Personality changes and difficulties in identifying social emotions have also been described [12, 13]. Deficits in executive function have been consistently reported in SCA1, SCA2, SCA3 and SCA6 [14–20]. In addition, visual memory, visual attention and visuo-spatial reasoning impairments have been described in SCA3 [21, 22]. To the best of our knowledge, no studies have described the neuropsychological profile or clinical course of SCA7 patients in detail. Relatively few studies have described the cognitive changes over time in SCAs. One follow-up study involving 10 SCA2 patients over an 8.5 year interval demonstrated a significant decline in non-verbal memory functions [23]. However verbal memory and executive functions, which were impaired at baseline, did not further deteriorate. In another study involving 11 SCA3 patients over a 3.5 year interval, declines in verbal learning, verbal memory and visual memory were reported [24]. It is also worth noting that amongst the autosomal dominant cerebellar ataxias, SCA2 is most commonly associated with dementia [25, 26].

We have previously carried out preliminary cognitive characterisations of five common SCA groups using an identical battery of neuropsychological tests, designed to take into account the specific disabilities associated with ataxia [27, 28]. The findings of these studies indicated a relatively similar cognitive profile for SCA1, SCA7 and SCA6 patients; SCA1 and SCA6 groups had relatively intact profiles and only selective deficits were noted in SCA7, primarily affecting executive function. In contrast, the SCA2 and SCA3 patients showed the greatest cognitive impairments. Attention was impaired in some of the patients in all groups except the SCA7 group and frontal executive deficits were present in all but the SCA1 group. Memory impairments were noted in SCA3, SCA6, and SCA7. These studies also examined social cognition, using the Theory of Mind test, and found that SCA3 and SCA6 groups performed poorly, as did one SCA1 individual. However, SCA2 and SCA7 groups did not differ significantly in their performance to normal controls. The aim of the current study is to provide a longitudinal characterisation of the clinical and cognitive profiles in SCA1, SCA2, SCA3, SCA6, and SCA7 patients from the original cohorts using follow-up data.

## Methods

### Patients

Of the 23 genetically confirmed SCA1, SCA2, SCA3, SCA6 and SCA7 patients who completed testing at baseline in our previous studies, 13 were available to be assessed at follow-up. The sample included two patients with SCA1, two with SCA2, two with SCA3, four with SCA6, and three with SCA7. Patients underwent two sets of clinical and neuropsychological assessment (baseline, follow-up) at the National Hospital for Neurology and Neurosurgery, with a mean inter-assessment interval of 7.5 years (SD 0.92 years). Structural neuroimaging was performed at follow-up. In addition, allele expansions were retrieved from the Department of Neurogenetics at the National Hospital for Neurology and Neurosurgery. The study was approved by the joint research ethics committee of the National Hospital for Neurology and Neurosurgery and the Institute of Neurology and performed in accordance with the ethical standards prescribed by the 1964 Declaration of Helsinki. All patients gave informed consent before participating.

### Clinical assessment

Ataxia symptoms and signs were assessed by a neurologist using SARA. The scale consists of eight items related to (1) gait, (2) stance, (3) sitting, (4) speech disturbance, (5) finger chase, (6) nose-finger test, (7) fast alternating hand movements, and (8) heel-shin slide. Total scores range from 0 to 40, with a higher score indicating a more severe phenotype. Non-ataxia signs were assessed by a neurologist using the INAS. The inventory is a list comprising 30 items which are grouped into 16 non-ataxia signs including areflexia, hyperreflexia, extensor plantar response, spasticity, paresis, amyotrophy, fasciculations, myoclonus, rigidity, chorea, dystonia, resting tremor, sensory symptoms, brainstem oculomotor signs, urinary dysfunction and cognitive impairment. The presence of absence of each of these signs is attributed a numerical value of either 1 or 0, respectively, providing a total score out of 16. In addition, we extracted and reported scores for impaired visual acuity (item 25).

### Neuropsychological assessment

A battery of neuropsychological and social cognitive tests was administered to all patients. The cognitive battery assessed the following domains: current intellectual functioning, premorbid intellectual functioning, verbal and visual recognition memory, nominal functions, calculation perceptual functions and attention and executive functions. The specific tests used have been detailed in our previous studies [27, 28]. Similarly, impairments in tests were classified as before to maintain consistency. Tests were chosen which had minimal demands on upper limb functions to reduce the influence of ataxia symptoms. Social cognition was tested using the Theory of Mind Test devised by Blair & Cipolotti and adapted by Van Harskamp et al. [29, 30]. A description of this test can be found in our previous studies [27, 28]. Mood was assessed using the Patient Health Questionnaire (PHQ-9) which is a brief, reliable and valid measure of depression severity [31]. PHQ-9 scores greater or equal to 10 indicate depression and scores of 5, 10, 15, and 20 represent mild, moderate, moderately severe, and severe depression, respectively [32].

### Neuroimaging

Structural MRI data (1.5 and 3 T) were acquired at follow-up using T1-weighted, T2-weighted and FLAIR acquisitions. These data were analysed and scored according to severity of atrophy in the cerebellum, brainstem, cortical and subcortical regions by a neuroradiologist. No focal vascular, space-occupying or inflammatory lesions were identified in our dataset.

### Data

For clinical assessments, baseline scores and change in score over time will be presented as group averages for both the SARA and INAS. Neuropsychological assessments will be reported in terms of the number of patients that achieve a score below standardised thresholds, suggesting impairment. Specific assessment thresholds are displayed in Table 3. Together with MRI atrophy scores, clinical, neuropsychological and mood scores will be described in terms of basic trends across groups. Statistical correlations will not be calculated due to the limited sample size.

## Results

### Sample demographics

Demographic details including age at onset, age at baseline, disease duration and CAG repeat length are displayed for each group in Table 1. The SCA1 group were notably younger, and the SCA6 group notably older, than the remaining groups at baseline. Disease duration was lowest in the SCA1 group. The CAG repeat lengths were clustered within SCA groups. With the exception of one, all patients had an estimated premorbid level of functioning in the average or high-average range. Of note, one SCA3 patient was not assessed on several verbal tests due to English being a second language. Notable changes in comorbidity include one SCA6 patient and one SCA7 patient being diagnosed with depression between baseline and follow-up. No other psychiatric or neurological diagnoses had been made during the interval.

**Table 1:** Sample Characteristics

					CAG repeat length (range)	
Group	n	Age at onset (range)	Age at baseline (range)	Follow-up period	Normal allele	Expanded allele
SCA1	2	30.5y (30–31)	36y (36)	6.5y	30.5 (29–32)	50.5 (50–51)
SCA2	2	40.5y (37–44)	52.5y (50–55)	7y	21.5 (21–22)	41 (41)
SCA3	2	32.5y (25–40)	45y (37–50)	8y	16 (14–20)	72.3 (70–74)
SCA6	4	47.5y (28–59)	42.5y (41–65)	7y	12.3 (11–13)	22.5 (22–23)
SCA7	3	33.7y (29–38)	42.3y (38–50)	7y	13.7 (12–15)	56.3 (46–65)

All values represent the group average (mean). Range values are given in brackets

### Clinical assessments

**Fig. 1:**
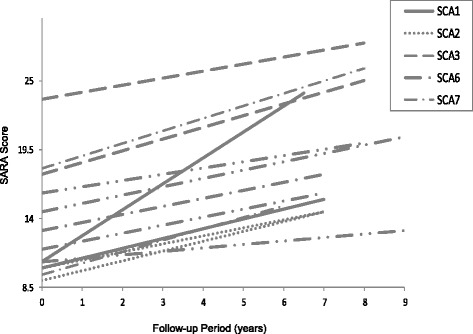
SARA scores over time for each patient

Baseline and follow-up scores for SARA and INAS are displayed in Table 2. Figure 1 shows longitudinal change in SARA score over time or each patient. All patients exhibited an increase in SARA score over time, representing longitudinal decline. Group average changes in SARA score ranged from 4.25 to 9.5. Patients in the SCA3 group were most impaired at baseline, whilst those in the SCA2 group were least impaired. The greatest increase in SARA score was observed in the SCA1 group. The remaining groups showed comparable decline over time, with patients in the SCA6 group showing the smallest change in score. The INAS scores increased in all groups over time. Group average changes in INAS score ranged from 0.5 to 4.5. Patients in the SCA1 group were most impaired at baseline, whilst those in the SCA6 group were least impaired. The greatest increase in INAS score was observed in the SCA3 group. The SCA2 and SCA6 groups showed minimal change in INAS score over time. Of note, INAS scores detected impaired visual acuity in all SCA7 patients, both at baseline and follow-up.

**Table 2:** SARA and INAS scores for each patient at baseline and follow-up

	SARA			INAS		
Patient	Baseline	Follow-up	Change	Baseline	Follow-up	Change
SCA1	10	15.5	+5.5	5	6	+1
SCA1	10.5	24	+13.5	3	5	+2
SCA2	10	14.5	+4.5	5	5	0
SCA2	9	14.5	+5.5	2	3	+1
SCA3	23.5	28	+4.5	3	6	+3
SCA3	17.0	2153	+7.5	3	9	+6
SCA6	14.5	20.5	+6	1	1	0
SCA6	11.5	16	+4.5	1	1	+2
SCA6	10.5	13	+2.5	0	2	+2
SCA6	16	20	+4	0	1	+1
SCA7	18	26	+8	3	5	+2
SCA7	13	17.5	+4.5	3	5	+2
SCA7	9.5	15	+5.5	2	3	+1

### Neuropsychological assessment

In general, calculation functions, nominal functions and perceptual functions were preserved in all groups both at baseline and follow-up. Performance IQ deficits were noted in each group except the SCA3 group, whilst verbal IQ deficits were only observed in the SCA1 and SCA6 groups.

Table 3 shows executive function, speed, attention and recognition memory data at baseline and follow-up, reported as number of patients within each group that were rated as impaired according to standardised threshold scores. The SCA1 group was the only group to exhibit decline in both verbal and visual recognition memory over time. The SCA6 group demonstrated no deficit in recognition memory, either at baseline or follow-up. Tests of speed, attention and executive function revealed deficits in all groups, albeit to varying degrees. Table 4 shows the scores for each patient on the Theory of Mind comprehension task. The SCA1, SCA2 and SCA7 groups were just above the threshold of impairment at baseline, whereas at follow-up, all groups had scored below threshold, again to varying degrees. The SCA7 group demonstrated the smallest decrease in score over time. In light of comprehension impairments, further comparison of the mental and physical justifications was not deemed appropriate (Additional file 1).

**Table 3:** Impairment on neuropsychological assessments in each group

	SCA 1 (*n* = 2)		SCA2 (*n* = 2)		SCA3 (*n* = 2)		SCA6 (*n* = 4)		SCA7 (*n* = 3)	
	Baseline	Follow-up	Baseline	Follow-up	Baseline	Follow-up	Baseline	Follow-up	Baseline	Follow-up
Executive Function										
Verbal Fluency FAS (<10th%ile)	0	1	0	0	1	2	0	0	1	1
Hayling Test (scaled score <3)	0	0	1	1	1	1	2	2	2	1
Stroop C-W Test (<5th%ile)	0	2	0	1	1	1	0	1	2^a^	1^a^
Speed and Attention										
Oral Symbol Digit Modalities Test (<5th%ile)	0	2	1	1	1	2	1^a^	1^a^	2	2
Elevator Counting with Distraction (<5th%ile)	1	1	1	1	1	0	0	0	0	0
Elevator Counting (score <5)	0	0	0	0	0	1	0	0	0	0
Recognition Memory										
RMW (<5th%ile)	0	1	0	1	0	0	0	0	0	0
RMF (<5th%ile)	0	2	0	0	1	2	0	0	1	2

Values displayed are number of patients that were impaired on each task, with specific thresholds in brackets (based on normalised scores). RMW/RMF = Recognition Memory Test, Words/Faces. ^a^means some data missing

**Table 4:** Theory of mind comprehension scores for each patient

Patient	Baseline score	Follow-up score	Change over time
SCA1	14	9^a^	−5
SCA1	14	13^a^	−1
SCA2	13^a^	13^a^	0
SCA2	14	14	0
SCA3	14	12^a^	−2
SCA3	12^a^	12^a^	0
SCA6	14	14	0
SCA6	12^a^	6^a^	−6
SCA6	14	13^a^	−1
SCA6	12^a^	11^a^	−1
SCA7	14	14	0
SCA7	14	13^a^	−1
SCA7	14	14	0

^a^indicates scores that are ≥2 standard deviations below healthy control group cores

### Neuroimaging

**Fig. 2:**
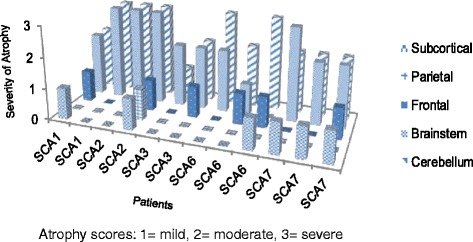
Regional atrophy on MRI brain in each SCA patient

MRI data are displayed in Fig. 2. Imaging was available for 12 of 13 patients at follow-up (one SCA6 patient did not complete an MRI visit). Cerebellar atrophy was ubiquitous, with all but two SCA3 patients exhibiting moderate or severe atrophy. Brainstem atrophy was observed in 11 of 12 patients, and this was moderate or severe in all but two cases. The SCA1 and SCA2 patients exhibited the greatest degree of cerebellar and brainstem atrophy. Cerebellar atrophy without brainstem atrophy was reported in one SCA6 patient. Subtle linear sagittal hyperintensities in the midline of the pons were reported in nine patients, involving all SCA groups. Mild frontal cortex atrophy was observed in six patients, involving all SCA groups. Mild parietal cortex atrophy was reported in one SCA2 patient. There was no occipital or temporal cortex atrophy noted within our sample. Mild subcortical atrophy was observed in all SCA7 patients.

### Mood

The PHQ-9 scores indicated that no patients met criteria for severe depression at baseline or follow-up. However, one SCA6 patient met criteria for major depression at baseline and follow-up but with little change over time. Of the remaining patients, one SCA1 and one SCA3 patient had a mild level of depression at baseline. At follow-up, the SCA3 patient continued to have mild depression while the SCA1 patient improved and no longer met this criterion. All other patients tested were below the cut-off for depression.

## Discussion

This study provides a preliminary characterisation of longitudinal change in both clinical and cognitive profiles in a small group of patients with SCA1, SCA2, SCA3, SCA6 and SCA7. To our knowledge, there has yet to be a follow-up study that incorporates both clinical and neuropsychological progression in these SCA patients, involving a follow-up period of this size. We used an identical battery of assessments to those used in previous studies, which has allowed for a reliable comparison of performance at both points in time [27, 28].

The SARA scores demonstrated progressive decline in ataxia symptoms in all patients. Despite having the shorter disease duration and shorter follow-up period, the SCA1 group exhibited the greatest increase in SARA over time, consistent with more rapid deterioration noted in this group of patients [6]. Whilst baseline INAS scores were highest in SCA1, the decline over time was not the greatest seen in our sample, as might be expected. Nevertheless, a pattern of decline involving both cerebellar and non-cerebellar domains was observed, consistent with the moderate to severe cerebellar and brainstem atrophy observed in this group [7]. The baseline SARA scores in the SCA3 patients were notably greater than the other groups, which might be in part attributable to the relatively early onset of disease and to the greater CAG expansion lengths observed in this group. In addition, the SCA3 group demonstrated the greatest increase in INAS score over time, reflecting the high frequency of non-ataxia features described in this subtype [6, 33]. The SCA2 group displayed the least decline in SARA and INAS scores over time when compared to the SCA1 and SCA3 groups. This pattern of SCA2 as the least severe phenotype amongst the type I autosomal dominant cerebellar ataxias is consistent with previous evidence [6, 26]. Interestingly, cerebellar and brainstem atrophy were rated as severe in both SCA2 patients. This is in line with previous quantitative imaging evidence that indicates a lack of correlation between infratentorial volume loss and neurological deficit in SCA2 [34]. The SCA6 patients exhibited the least decline on SARA and only mild decline on INAS, consistent with the slower and non-linear decline described in this sub-type [6]. Although the relatively low INAS scores in this group might support the traditional notion of SCA6 being a “pure cerebellar ataxia”, MRI findings demonstrated brainstem atrophy in two of three patients and frontal cortex atrophy in two of three patients. It should be noted that, consistent with the later onset reported in SCA6, the patients in this group were older than the majority of other patients in our sample. It is thus important to include the possibility of the effects of ageing when trying to account for this pattern of performance and atrophy. The SCA7 group demonstrated moderate decline on SARA and INAS scores over time, despite a shorter disease duration than most. Expanded allele CAG repeat lengths in this group were greater than in the SCA1, SCA2 and SCA6 groups, offering some explanation for the younger age at onset and the more marked clinical progression in these patients [35]. It is also important to note the presence of visual acuity impairments in all of the SCA7 patients at baseline, consistent with the typical phenotype in this subgroup. Mild subcortical atrophy was observed in all SCA7 patients, which could be associated with the cerebro-cerebellar atrophy known to correlate with ataxia severity in these patients [36].

The SCA1 patients exhibited the greatest degree of impairment over time on neuropsychological testing, congruent with the motor deterioration seen in this subtype. In particular executive function, speed and attention deteriorated from normal scores at baseline. This pattern of decline raises the question as to whether there exists a common patho-anatomical basis to the motor and cognitive manifestations of SCA1. Volumetric analyses have noted a correlation between brainstem atrophy and motor symptoms in SCA1, and so it would be interesting to investigate whether infratentorial volume has any direct impact on cognitive performance in these patients [7, 12, 37]. The decline in visual memory in the SCA1 patients is not consistent with another longitudinal study, however the follow-up period in the present study was significantly greater [14]. Although there is little evidence describing the cognitive profile of murine models of the SCAs, it is worth noting that severe deficits in visuospatial functions, as assessed using the spatial version of the Morris water maze, have been reported in transgenic SCA1 mice [38]. Furthermore, these deficits were associated with diminished paired-pulse facilitation in hippocampal CA1 neurons, implicating medial temporal lobe pathology in the cognitive profile of these mouse models. Decline in visual memory was also observed in SCA3 patients, which is consistent with previous reports of poor performance on tasks of visual memory, visual attention and visuospatial functions [21, 22]. Interestingly, a transgenic SCA3 mouse model exhibited normal performance on the Morris water maze, perhaps highlighting the often challenging translation of such measures between murine models and patients [39]. In the tasks of executive function, speed and attention, the SCA3 patients also exhibited decline although deficits were already more pronounced at baseline. Whereas the pattern of cognitive decline seen in the SCA1 and SCA3 groups was loosely comparable, their clinical scores were quite different: the SCA1 patients showed a lower SARA score but a higher INAS score compared to the SCA3 group, and it was the latter group that exhibited the more pronounced change on the INAS over time. When considered in the context of the more severe atrophy noted in the SCA1 group, these results might suggest that the cognitive decline observed in these patients is not entirely attributable to the infratentorial changes seen on MRI. The SCA2 group displayed the more stable neuropsychological profile over time, which is consistent with another longitudinal study involving a similar follow-up period [22]. Although impairments were noted in this group on tasks of speed, attention and executive function, relatively little change over time was observed. Ataxin-2 knockout mice displayed normal visuospatial functions and associated intact hippocampal long-term potentiation, providing some suggestion of unaffected memory functions in these models [40]. In the motor domain, the SCA2 patients exhibited mild decline in SARA and INAS scores, suggesting a generally less severe phenotype. The severe cerebellar and brainstem atrophy in this group is consistent with the suggestion that early disruption to cerebro-cerebellar pathways yields a non-progressive “frontal-executive dysfunction” syndrome in SCA2 [16, 23]. The SCA6 patients were less impaired overall compared to the other groups, in line with the milder progression of motor symptoms observed in this group. Some decline was noted, specifically in executive functioning, speed, attention and performance IQ, where the latter has not previously been demonstrated [20, 41]. The presence of mild frontal cortex atrophy in two of these patients may also have contributed to these deficits, which in turn must be considered in the context of age; the SCA6 patients were among the oldest in our sample and so potential age-related changes in cortical structure should not be overlooked [42]. The SCA7 patients demonstrated decline on the tests of speed, attention and executive function, and this group were notably more impaired at baseline. Importantly, we used aural assessments in all domains thus controlling for any visual deficits in this group. The SCA7 patients were impaired on both verbal and visual measures of executive function. Interestingly, only the visual tests of speed and attention yielded abnormal scores, whilst the verbal tests revealed no or little impairment. It is therefore possible that speed and attention are not necessarily affected in SCA7, as revealed by tests that don’t rely on visual input. This group did exhibit decline on tasks involving visual memory, performance IQ and visual-perceptual functions, as would be expected [43, 44]. Mild subcortical atrophy was noted in each SCA7 patient, however the relevance of this to the cognitive profile described is difficult to ascertain. Theory of Mind comprehension scores revealed deficits in most patients at follow-up, and no obvious group differences were detected. Impairments in social cognition thus appear to be an important component of the SCA profile, which might not be associated with clinical or other neuropsychological measures [45].

In the current study, no correlations between clinical decline and mood scores were observed. Furthermore, cognitive decline and mood scores were not related. Indeed, a number of patients exhibited improvements in depression ratings whilst deteriorating on neuropsychological testing. Two patients were taking antidepressants during the study follow-up period and despite medication, PHQ-9 scores remained static. It is thus felt that medications had an insignificant effect on mood scores in our study. In light of the sample size it is difficult to extrapolate these results, but a similar lack of association between mood, clinical and cognitive scores has been noted previously in SCA patients [46]^.^

The numbers of patients within each SCA subtype group were small, making comparisons difficult. Our findings are therefore very preliminary and only larger studies will be able to confirm our findings. However, despite the sample size of our cohort, many of our findings seem to fit with previous research suggesting different patterns of cognitive decline according to SCA subtype. We believe that this is due in part to the viability of our neuropsychological battery designed specifically for ataxia patients. The use of the SARA and INAS for physical symptoms is also consistent with previous research in a large cohort of patients [47]. MR imaging was only performed at follow-up, and so we have been unable to comment on progressive change in brain structure in these patients. Indeed, longitudinal imaging data should be used in future studies in order to help clarify any anatomical correlates of cognitive function in the SCAs. Our use of the PHQ-9 may have also limited our findings on mood as this does not extensively examine both anxiety and depression.

## Conclusion

Cognitive decline seems to be a distinct aspect of the SCA phenotype, independent of mood disturbance. Specifically, tests of executive function, speed and attention were most sensitive to change over time in the majority of our sample. We have identified a trend between cognitive and clinical decline in specific SCA subtypes. The SCA1 and SCA3 patients exhibited the more severe cerebellar impairments (SARA) together with the greatest cognitive decline over time. In contrast, the SCA2 and SCA6 groups demonstrated the least decline on both cerebellar and cognitive assessments. This pattern of motor impairment amongst SCA subtypes has recently been confirmed in a large observational study [47]. Cognitive performance does not seem to be associated with the cerebellar impairments observed in SCA7. Furthermore, the cognitive profile seems independent of the extra-cerebellar manifestations of disease in all studied patients as measured by the INAS. We were unable to identify MRI indices of atrophy that might explain the different patterns of cognitive impairment detected in SCA subtypes. The cognitive profiles we report here adhere to the previously described “Cerebellar Cognitive-Affective Syndrome” originally described in patients with focal cerebellar lesions [12]. Studies involving large samples and longitudinal imaging protocols will help to elucidate further the patho-anatomical basis of different neuropsychological impairments in different SCA subtypes, and in so doing contribute to our understanding of the role of the cerebellum in cognition.

### Ethics approval and consent to participate

The study was approved by the joint research ethics committee of the National Hospital for Neurology and Neurosurgery and the Institute of Neurology (REC number 04/Q505/21) and performed in accordance with the ethical standards prescribed by the 1964 Declaration of Helsinki. All patients gave informed consent before participating.

### Consent for publication

Not applicable.

### Availability of data and supporting materials

This submission includes all raw data for the SARA, INAS and cognitive assessments in full.

## Additional file

Additional file 1: Neuropsychological assessments, raw scores I & II. Data displayed as raw scores for each patient on each assessment, at baseline and follow-up. Greyed scores represent impairment based on normalised scores thresholds. CW = colour-word; RMW/RMF = Recognition Memory Test, Words/Faces. ~ means data missing. (PDF 63 kb)

## Abbreviations

INAS: Inventory of non-ataxia signs
PHQ-9: Patient health questionnaire
SARA: Scale for the rating and assessment of ataxia
SCA: Spinocerebellar ataxia
SD: Standard deviation
