# Supplementary material for: A longitudinal investigation into cognition and disease progression in spinocerebellar ataxia types 1, 2, 3, 6, and 7
Source: Orphanet J Rare Dis. 2016 Jun 22;11:82. doi: 10.1186/s13023-016-0447-6 (PMC4917932; doi:10.1186/s13023-016-0447-6)
Supplement: Additional file 1: — Neuropsychological assessments, raw scores I & II. Data displayed as raw scores for each patient on each assessment, at baseline and follow-up. Greyed scores represent impairment based on normalised scores thresholds. CW = colour-word; RMW/RMF = Recognition Memory Test, Words/Faces. ~ means data missing. (PDF 63 kb) [file 13023_2016_447_MOESM1_ESM.pdf]

Neuropsychological assessments, raw scores I: executive function

ID	SCA	Executive Function							
		Verbal Fluency FAS		Hayling Sentence Competion				Stroop CW	
		Baseline	Follow-up	Baseline raw	Baseline scaled	Follow-up raw	Follow-up scaled	Baseline	Follow-up
1	1	41	23	18	6	15	5	112	81
2	1	32	26	18	6	17	6	108	67
3	2	41	36	19	6	16	5	68	49
4	2	43	49	10	2	11	3	81	64
5	3	15	9	11	3	12	3	72	45
6	3	32	20	15	5	13	4	112	105
7	6	34	31	11	3	17	6	84	57
8	6	31	23	11	3	12	3	82	80
9	6	27	26	15	5	13	4	79	74
10	6	32	34	16	5	9	1	93	84
11	7	27	31	12	3	13	4	49	~
12	7	26	17	9	1	7	1	~	~
13	7	41	35	18	6	17	6	34	32

Neuropsychological assessments, raw scores II: speed and attention, recognition memory

ID	SCA	Speed and Attention						Recognition Memory			
		Symbol Digit		Elevator Counting		Elevator Counting with distraction		RMW		RMF	
		Baseline	Follow-up	Baseline	Follow-up	Baseline	Follow-up	Baseline	Follow-up	Baseline	Follow-up
1	1	46	33	7	7	10	5	42	41	42	36
2	1	44	31	7	7	2	3	40	30	41	30
3	2	31	30	7	7	7	7	49	48	39	37
4	2	36	33	7	7	5	4	40	28	39	39
5	3	33	26	7	5	3	6	44	39	36	35
6	3	50	32	7	7	10	9	48	46	38	37
7	6	33	20	7	7	4	4	50	43	41	45
8	6	36	30	7	7	10	9	47	44	47	45
9	6	~	~	7	7	5	5	50	48	46	48
10	6	44	39	7	6	4	3	47	23/25	48	25/25
11	7	32	28	7	6	10	5	49	44	41	29
12	7	23	22	7	6	7	6	50	44	33	28
13	7	28	22	6	7	8	8	50	50	45	42
